# α-cell glucokinase suppresses glucose-regulated glucagon secretion

**DOI:** 10.1038/s41467-018-03034-0

**Published:** 2018-02-07

**Authors:** Davide Basco, Quan Zhang, Albert Salehi, Andrei Tarasov, Wanda Dolci, Pedro Herrera, Ioannis Spiliotis, Xavier Berney, David Tarussio, Patrik Rorsman, Bernard Thorens

**Affiliations:** 1 Center for Integrative Genomics, University of Lausanne, 1015 Lausanne, Switzerland; 2 Oxford Centre for Diabetes, Endocrinology, and Metabolism, University of Oxford, Churchill Hospital, Oxford, OX3 7LE UK; 3 Department of Clinical Science, UMAS, Division of Islet Cell Physiology, Lund, Sweden; 4 Department of Genetic Medicine and Development, 1200 Geneva, Switzerland

## Abstract

Glucagon secretion by pancreatic α-cells is triggered by hypoglycemia and suppressed by high glucose levels; impaired suppression of glucagon secretion is a hallmark of both type 1 and type 2 diabetes. Here, we show that α-cell glucokinase (*Gck*) plays a role in the control of glucagon secretion. Using mice with α-cell-specific inactivation of *Gck* (*αGckKO* mice), we find that glucokinase is required for the glucose-dependent increase in intracellular ATP/ADP ratio and the closure of K_ATP_ channels in α-cells and the suppression of glucagon secretion at euglycemic and hyperglycemic levels. *αGckKO* mice display hyperglucagonemia in the fed state, which is associated with increased hepatic gluconeogenic gene expression and hepatic glucose output capacity. In adult mice, fed hyperglucagonemia is further increased and glucose intolerance develops. Thus, glucokinase governs an α-cell metabolic pathway that suppresses secretion at or above normoglycemic levels; abnormal suppression of glucagon secretion deregulates hepatic glucose metabolism and, over time, induces a pre-diabetic phenotype.

## Abstract (web summary)

Glucagon secretion is promoted during hypoglycemia and inhibited by increased glucose levels. Here, Basco et al. show that glucokinase suppresses glucose-regulated glucagon secretion by modulating the intracellular ATP/ADP ratio and the closure of K_ATP_ channels in α-cells.

## Introduction

Glucagon secretion by pancreatic α-cells is rapidly increased when the blood glucose concentration falls below the normoglycemic level to increase hepatic glucose production, and is suppressed by hyperglycemia^1,2^. The mechanisms controlling hypoglycemia-induced glucagon secretion remain debated, and both intrinsic and paracrine mechanisms have been postulated (reviewed in refs. ^3,4^). There is evidence that hypoglycemia triggers glucagon secretion via a fall in the cytoplasmic ATP/ADP ratio, leading to moderate K_ATP_ channel activity and increased activity of P/Q type Ca^++^ channels^3^. The resulting increase in intracellular Ca^2+^ leads to glucagon secretory granules exocytosis. Extrinsic factors also play an important role in triggering glucagon secretion, in particular, the signals from the sympathetic and parasympathetic branches of the autonomic nervous system^4,5^, which are activated by hypoglycemia-sensing neurons present in the extrapancreatic sites, such as the hepatoportal vein area^6,7^ and the central nervous system^5,8,9^. On the other hand, suppression of glucagon secretion by hyperglycemia relies on paracrine regulation, including insulin-induced inhibition and/or somatostatin-induced inhibition of α-cells^10^.

In pancreatic β-cells, the dose response of glucose-stimulated insulin secretion is controlled by the activity of glucokinase (*Gck*), which has relatively high *K*_M_ for glucose (8 mM)^11^. Mutations that increase the K_M_ for glucose of this enzyme shifts the dose response of GSIS to the right, causing Maturity Onset Diabetes of the Young 2 (MODY2)^12^. Glucokinase is also expressed in α-cells^13^, but its role in the control of glucagon secretion is not known. Studies of MODY2 patients during stepped hyperinsulinemic/hypoglycemic clamps showed that glucagon secretion was stimulated at higher glucose concentration than in control individuals, but whether this was due to a direct effect in the α-cells or secondary to altered glucose-sensing by the central nervous system and altered autonomic nervous activity remains unclear^14^. Here we explore the role of *Gck* in the pancreatic α-cell by generating α-cell-specific *Gck* knockout mice. Our data illustrate that Gck is critical to glucose sensing in the α-cell and underscore the significance of intrinsic (exerted within the α-cell itself) as opposed to paracrine/systemic regulation.

## Results

### Characterization of *αGckKO* islets

**Fig. 1:**
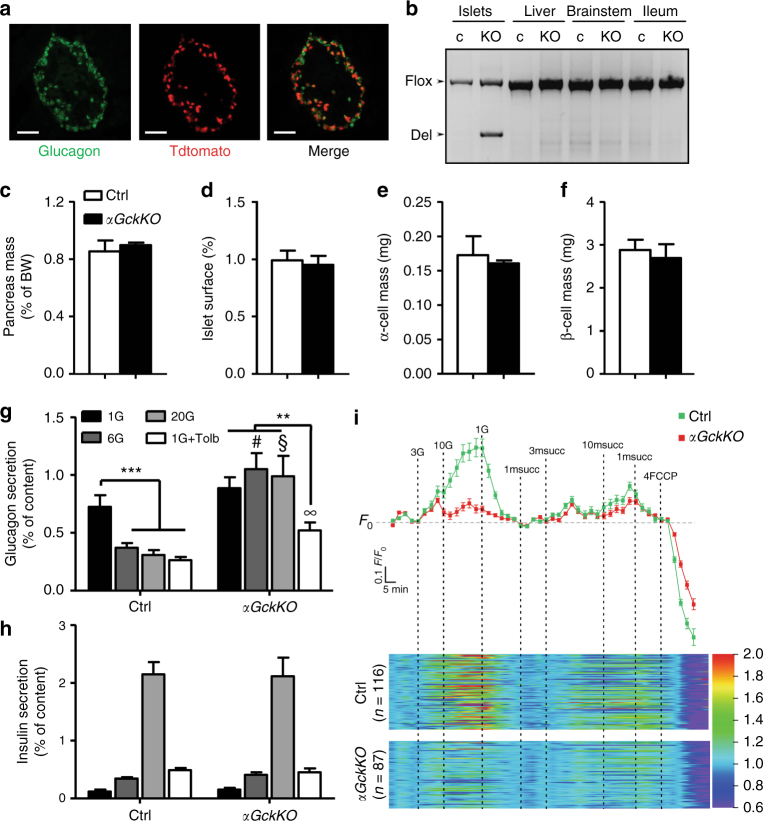
Alpha-cell *Gck* inactivation and the suppression of glucagon secretion. **a** Representative immunofluorescence (out of *n* = 3 replicates) of the co-localization of glucagon (green) and tdtomato (red) in a pancreatic section from *αGckKO-Rosa26tdtomato* mice. Scale bar: 100 µm. **b** PCR analysis of recombination of the Gck^flox^ allele in the indicated tissues of Ctrl and *αGckKO mice*. **c** Pancreas' weight normalized to BW. See also Supplementary Fig. 1. **d** Islet surface expressed as percentage of total pancreatic surface. **e** α-cell mass. **f** β-cell mass. Values in **b**–**h** were extracted from the analysis of three pancreata for each genotype. **g** Glucagon and **h** Insulin secretion from isolated islets in the presence of the indicated glucose (G) concentrations and of tolbutamide. **g**, **h** Data represent the average of three independent experiments, each performed in duplicates. ***p < 0.001 vs. Ctrl 1G; **p < 0.01 vs. *αGckKO* 1G + Tolb. #*p* < 0.01 vs. Ctrl 6 G. §*p* < 0.001 vs. Ctrl 20G. **∞***p* < 0.05 vs. Ctrl 1G + Tolb. One-way ANOVA for intra-group analysis and Student’s *t*-test for comparison between genotypes. **i** ATP/ADP ratio measurements in α-cells of Ctrl and *αGckKO* islets exposed to glucose and methyl-succinate (msucc). *n* = 116 for Ctrl and *n* = 86 for *αGckKO* α-cells. See also Supplementary Figs. 2 and 3. Data are represented as mean ± s.e.m.

To generate mice with inactivation of the *Gck* gene in α-cells (*αGckKO* mice), we crossed *Gck*^*lox/lox*^ mice^9^ with *preproglucagon-Cre* (*Gcg-Cre*) mice^15^. These mice were further crossed with *Rosa26-tdtomato* mice and ~70% of the glucagon-positive cells also expressed tdtomato (Fig. 1a), indicating that a large majority of α-cells express the Cre recombinase. The recombined *Gck* allele was detected in islets of *αGckKO* mice, but not in their liver, brainstem, and ileum tissues that also express the preproglucagon gene, but not the Cre recombinase in the *Gcg-Cre* mice utilized (Fig. 1b). Pancreas mass, islet surface area, α-cell mass and β-cell mass (Fig. 1c–f), as well as pancreatic insulin and glucagon contents (Supplementary Fig. 1) were the same in Ctrl and *αGckKO* mice.

The impact of α-cell *Gck* gene inactivation on glucagon secretion was then examined by static incubations. At 1 mM glucose, glucagon secretion by islets from 18-week-old Ctrl and *αGckKO* mice was comparable (Fig. 1g, black bars). When incubated with 6 and 20 mM glucose, glucagon release by Ctrl islets was decreased by ~50%, but not in *αGckKO* islets (Fig. 1g). Tolbutamide, which closes the K_ATP_ channel independently of glucose metabolism and changes in the ATP/ADP ratio, produced a comparable inhibition of glucagon secretion in both types of islets when applied at 1 mM glucose (Fig. 1g, white bars). Insulin secretion by Ctrl and *αGckKO* islets was similarly stimulated by increases in glucose concentrations (Fig. 1h). Thus, although *Gck* is not required for the high rate of glucagon secretion at 1 mM glucose, it is critical to the suppression produced by elevated glucose.

### Suppressed glucose-induced ATP production in *αGckKO* α-cells

To assess whether *Gck* inactivation prevented ATP production in the presence of elevated extracellular glucose concentrations, we measured the intracellular ATP/ADP ratio in Ctrl and *αGckKO-Rosa26tdtomato* α-cells transduced with a recombinant adenovirus to express the Perceval reporter protein^16^. Perceval fluorescence in tdtomato-expressing α-cells was measured by confocal microscopy in the presence of different glucose concentrations (Fig. 1i). In control α-cells, the ATP/ADP ratio increased in the presence of 3 and 10 mM glucose, an effect that was fully reversible upon return to 1 mM glucose. The glucose-induced increase in cytoplasmic ATP/ADP-ratio was absent in α-cells from *αGckKO* mice. However, application of methyl-succinate (msucc), a cell-permeable substrate of the tricarboxylic cycle, produced similar increment in the ATP/ADP ratio in Ctrl and *αGckKO* α-cells. In a separate experiment, we measured the ATP/ADP ratio at 1, 3, and 6 mM glucose. Over this narrower range of glucose concentrations, we observed a clear increase in the ATP/ADP ratio already at 3 mM glucose in Ctrl α-cells, but not in α-cells from *αGckKO* mouse islets (Supplementary Fig. 2). Glucose-induced changes in the ATP/ADP ratio in β-cells from *αGckKO* islets were identical to those in control islets (Supplementary Fig. 3).

### Electrophysiology in *αGckKO* islets

**Fig. 2:**
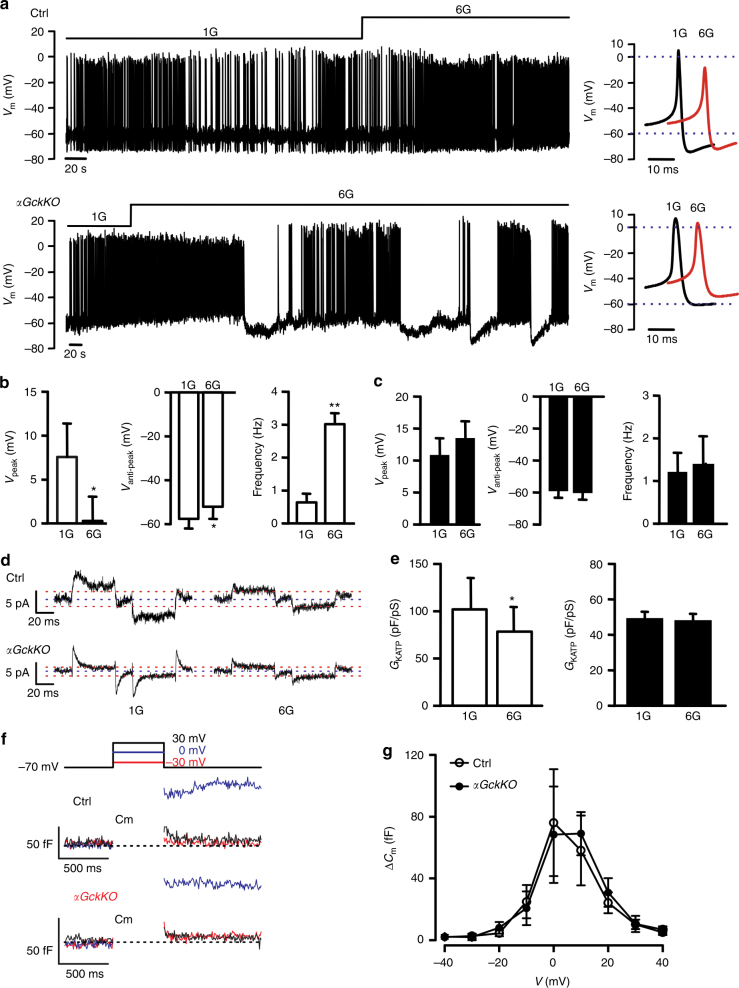
Electrophysiology of *αGckKO* α-cells. **a** Examples of α-cells electrical activity in response to the indicated glucose concentration recorded from Ctrl (top line) and *αGckKO* islets (bottom line). Right panels: single action potentials recorded at 1G (black) or 6G (red) with expanded time scale. See also Supplementary Fig. 4. **b** Summary of Ctrl α-cell action potential peak voltage, anti-peak voltage, and firing frequency in response to 1G and 6G glucose. *n* = 4; *p < 0.05; Student’s *t*-test. **c** Same as **b** for *αGckKO* α-cells. *n* = 4. **d** Examples of K_ATP_ current measured in α-cells of Ctrl (upper panel) and *αGckKO* (lower panel) islets. **e** Summary of K_ATP_ conductance, expressed as delta between 1G and 6G glucose (normalized to cell size; pS/pF), measured at 1G and 6G. *n* = 6 per genotype. *p < 0.05; Student’s *t*-test. Data are represented as mean ± s.d. **f** Voltage-dependent exocytosis in α-cells of Ctrl (middle traces) and *αGckKO* (bottom traces) islets. Cell exocytosis was monitored as increase in cell membrane capacitance (ΔCm) induced by membrane depolarizations. The responses were evoked by depolarizations from −70 mV to the indicated voltages (top schematic traces: red −30 mV, blue 0 mV  + 30 mV). **g** Relationship of the α-cell exocytosis (ΔCm) with depolarizations from −70 mV to different voltages (*V*). Open circles Ctrl (*n* = 10), black filled circles *αGckKO* (*n* = 6). Data are represented as mean ± s.e.m.

We next performed electrophysiological analysis of α-cells in Ctrl and *αGckKO* islets. At 1 mM glucose, α-cells from Ctrl and *αGckKO* islets were electrically active and fired overshooting action potentials that peaked at 8 ± 4 mV (*n* = 5) and 11 ± 3 mV (*n* = 4) for Ctrl and *αGckKO* α-cells, respectively (Fig. 2a). In agreement with previous data, increasing glucose to 6 mM resulted in a small and reversible membrane depolarization (+6 ± 2 mV, *n* = 5) and an ~10 mV reduction of the action potential peak voltage in α-cells from Ctrl islets. By contrast, increasing glucose neither depolarized nor reduced the action potential peak voltage in *αGckKO* α-cells (Fig. 2a–c). In *αGckKO* α-cells, glucose, if anything, tended to hyperpolarize the α-cell and increase action potential height. These effects may reflect somatostatin release from the neighboring δ-cells^17^.

In Ctrl α-cells, increasing glucose reduced the resting K_ATP_ conductance by 24 ± 11pA/pF; no such decrease was observed in *αGckKO* α-cells (Fig. 2d, e).

We ascertained (using capacitance measurements^3^) that depolarization-evoked exocytosis was the same in Ctrl and *αGckKO* α-cells: in both cell types, membrane depolarization to 0 mV produced step increases in membrane capacitance (Fig. 2f). The voltage dependence of exocytosis in α-cells is summarized in Fig. 2g. Exocytosis was steeply voltage-dependent at membrane potentials between −20 and + 10 mV.

We performed electrophysiological analysis of glucose-induced electrical activity in β-cells from adult *αGckKO* and Ctrl mice. When exposed to stimulatory concentrations of glucose (10 or 20 mM), β-cells of both genotypes were depolarized and fired bursts of action potentials (Supplementary Fig. 4).

### Hyperglucagonemia and gluconeogenesis in *αGckKO* mice

**Fig. 3:**
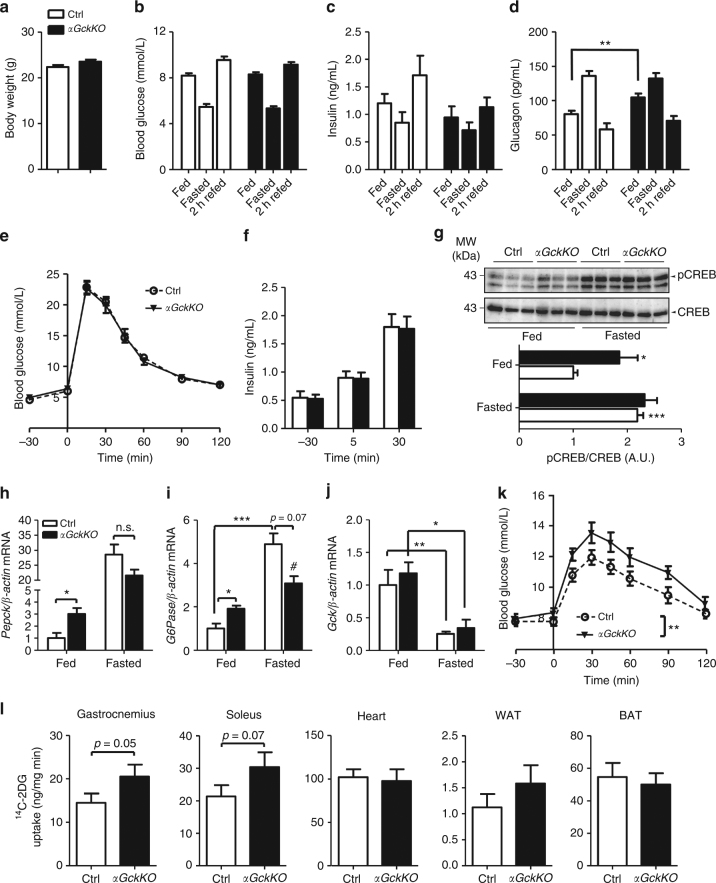
Increased hepatic glucose production in *αGckKO* mice. **a** Body weight, **b** Blood glucose, **c** Plasma insulin, and **d** Plasma glucagon. **a**–**d** 20 week-old female mice. *n* = 19 Ctrl and *n* = 27 *αGckKO* mice; **p < 0.01. Student’s *t*-test. See also Supplementary Fig. 5. **e** Intra-peritoneal glucose tolerance test. *n* = 13 mice for each genotype. **f** Plasma insulin at the indicated times before and after i.p. glucose injection. *n* = 13 mice for each genotype. **g** CREB and phosphorylated CREB in fed and fasted Ctrl and *αGckKO* mouse livers. See also Supplementary Fig. 8. **h** *Pepck*, **i** *G6Pase* and **j** *Gck* mRNA levels in the livers from fed and fasted Ctrl and *αGckKO* mice. **h**–**j** *n* = 4–5 per genotype; *p < 0.05, **p < 0.01, ***p < 0.001, ^#^*p* < 0.05 vs. Ctrl fed state; Student’s *t*-test. See also Supplementary Fig. 6 and Supplementary Table 1. **k** Pyruvate tolerance test. *n* = 11 mice per genotype; **p < 0.01 (Two-way ANOVA). **l** ^14^C-2DG uptake in the indicated tissues of Ctrl and *αGckKO* mice. *n* = 9 Ctrl and *n* = 10 *αGckKO* mice. *p < 0.05; Student’s *t*-test. Data are represented as mean ± s.e.m.

Eighteen to twenty weeks old female and male Ctrl and *αGckKO* mice had similar body weight (Fig. 3a and Supplementary Fig. 5a), blood glucose levels, and plasma insulin in fed, fasted, and fasted and 2-hours refed states (Fig. 3a–c for female mice and Supplementary Fig. 5a–c for male mice). Plasma glucagon levels were similar in fasted and 2-hours refed mice, but were 30% higher in the fed condition in female *αGckKO* mice (Fig. 3d). A similar trend was also found in *αGckKO* male mice (Supplementary Fig. 5d). Glucose tolerance determined after intraperitoneal (Fig. 3e) or oral (not shown) glucose administration was normal in female *αGckKO* mice, as was in vivo glucose-stimulated insulin secretion (GSIS) (Fig. 3e, f).

To determine whether the hyperglucagonemia observed in fed female mice was impacting liver glucose metabolism, we first measured phosphorylated cAMP response element–binding protein (pCREB) and the level of expression of phosphoenolpyruvate carboxykinase (*Pepck*) and glucose-6-phosphatase (*G6Pase*) mRNAs in fed and overnight fasted mice. Analysis of pCREB/CREB ratio showed that pCREB was two-fold higher in the fed state in the livers of *αGckKO* than that in Ctrl mice (Fig. 3g). Both *Pepck* and *G6Pase* mRNAs were expressed at a higher level in the liver of fed *αGckKO* mice as compared to Ctrl mice, whereas no significant differences were detected between genotypes in overnight fasted mice (Fig. 3h,i). The expression levels of *Gck* (Fig. 3j) and several fatty acid oxidation genes (*Mcad, Pgc-1α*, *Cpt-1*) and fatty acid synthesis (*Fas*, *Acc*) were also measured and found to be similar in the livers of Ctrl and *αGckKO* mice (Supplementary Fig. 6a–e).

To assess whether the higher expression of gluconeogenic genes in fed *αGckKO* mice was associated with increased gluconeogenic activity, we performed pyruvate tolerance tests. Fig. 3k shows that pyruvate administration led to significantly higher blood glucose levels in *αGckKO* mice as compared to Ctrl mice (*p* < 0.01). As enhanced hepatic glucose output in *αGckKO* mice was not associated with fed hyperglycemia (see Fig. 3b), we hypothesized that increased glucose disposal could maintain normal glycemic control. We thus performed in vivo radioactive 2-deoxy-glucose (^14^C-2DG) uptake assays to monitor glucose uptake in various tissues. ^14^C-2DG uptake was higher in skeletal muscle (gastrocnemius and soleus) of *αGckKO* mice as compared to Ctrl mice (Fig. 3i). No differences in glucose uptake were found in the heart, white and brown adipose tissues. Thus, fed hyperglucagonemia increased hepatic gluconeogenic gene expression and activity. Normal glycemic control was, however, preserved because of increased glucose uptake in skeletal muscles, which was, however, not associated with increased insulin sensitivity as measured by insulin tolerance test (Supplementary Fig. 6f).

### Hyperglucagonemia and prediabetes in adult *αGckKO* mice

**Fig. 4:**
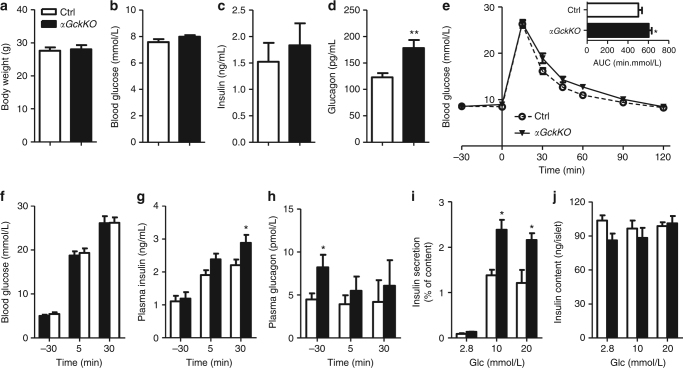
Prediabetic phenotype in 36-week-old *αGckKO* mice. **a** Body weight, **b** Blood glucose, **c** Plasma insulin, and **d** Plasma glucagon in 36-week-old, fed female mice (*n*  =  9 Ctrl and *n * =  13 *αGckKO mice*). **p < 0.01 vs. Ctrl. **e** I.p. glucose tolerance test. Inset: area under the curve (AUC). *p < 0.05 vs. Ctrl. Student’s *t*-test. **f** Blood glucose, **g** Plasma insulin, and **h** Plasma glucagon, at the indicated time before or after i.p. glucose injection. *p < 0.05 vs. relative Ctrl. **i** Glucose-stimulated insulin secretion from islets isolated from 36-week-old mice. *p < 0.05 vs. relative Ctrl. Student’s *t*-test. **j** Total insulin content from 36-week-old mouse islets. Data showed in **i**, **j** represent the average of three independent experiments, each performed in duplicate. See also Supplementary Fig. 7. Data are represented as mean ± s.e.m.

To evaluate whether the observed deregulations of glucagon secretion and liver metabolism can induce long-term impairment in glucose homeostasis, we studied 36-week-old Ctrl and *αGckKO* mice. No significant differences in body weight, fed blood glucose, and plasma insulin levels were detected between Ctrl and *αGckKO* mice levels (Fig. 4a–c). As was the case in younger mice, adult *αGckKO* female mice had a higher fed plasma glucagon level (+45%) than Ctrl mice (Fig. 4d), and developed mild glucose intolerance after an overnight fast (Fig. 4e). In in vivo glucose-stimulated insulin secretion experiments (after a 6-h fast), *αGckKO* female mice secreted significantly more insulin 30 min after i.p. glucose injection (Fig. 4g), despite similar blood glucose levels (Fig. 4f); their glucagonemia was also significantly elevated 30ʹ before i.p. glucose injection (Fig. 4h). To assess whether the increase in insulin secretion was caused by increased β-cell secretion capacity, we performed insulin secretion experiments using isolated islets. Fig. 4i shows that insulin secretion was markedly higher in islets from adult *αGckKO* mice as compared to those of adult Ctrl mice, despite identical islet insulin contents (Fig. 4j). To determine whether this could be due to increased glucagon secretion from α-cells or to increased processing of preproglucagon into, and secretion of GLP-1 from α-cells, we performed insulin secretion experiments in the presence of the glucagon receptor antagonist L-168,049 and of the GLP-1R antagonist exendin(9-39). No inhibitory effects on glucose-stimulated insulin secretion by islets from *αGckKO* mice could be observed in these conditions (Supplementary Fig. 7a, b); qRT-PCR analysis also showed that the level of *Pcsk1/3* was not increased in *αGckKO* α-cells (Supplementary Fig. 7c).

## Discussion

Here, we show that inactivation of *Gck* in α-cells results in hyperglucagonemia in the fed state and increased hepatic glucose production. In adult mice, these initial defects lead to a further increase in fed hyperglucagonemia and mild glucose intolerance. This is also associated with increased insulin secretion in response to glucose stimulation in vivo and in vitro, indicating that, over time, unsuppressed glucagon secretion induces a compensatory increase in insulin secretion.

It is now well established that stimulation of glucagon secretion depends on an α-cell intrinsic hypoglycemia detection system^3^ and on extrinsic signals, in particular, arising from increased autonomic nervous activity^4,8,18^. The dramatic effect of genetic ablation of Gck in α-cells on the capacity of glucose to suppress glucagon secretion while leaving glucose-sensing in the non-α-cells illustrates the importance of intrinsic (i.e., within the α-cell itself) regulation of glucagon secretion. A current model of the regulation of the α-cells postulates that an increase in (plasma) glucose inhibits glucagon secretion via an increase in intracellular ATP/ADP ratio and closure of ATP-regulated K^+^ channel, which results in membrane depolarization and voltage-dependent inactivation of the voltage-gated Na^+^ channels involved in action potential firing, leading to a diminution of action potential height and a decreased activation of the P/Q Ca^2+^ channels that mediate the Ca^2+^ entry, triggering glucagon release^19^. This mechanism is almost maximally activated at glucose concentrations not associated with any (major) stimulation of insulin and glucagon secretion^18^. We acknowledge that other mechanisms for intrinsic regulation of glucagon secretion have been proposed (reviewed by Gylfe; see ref. ^19^), and that the involvement of an intrinsic regulation does not negate the possibility that the α-cell is also under paracrine, hormonal, and neuronal control. Clearly, *Gck* in α-cells is a necessary component of the glucose-sensing apparatus. It is interesting that α-cells remain capable of electrical activity even after genetic ablation of *Gck*. Clearly, the cytoplasmic ATP/ADP ratio remains sufficiently high in α-cells at low glucose and in the absence of activation of *Gck* to keep the K_ATP_ channels sufficiently closed to allow action potential firing. How this occurs remains unclear, but it is notable that α-cells express hexokinase 1 (*Hk1*), a high-affinity hexokinase. It will be interesting to explore the phenotype in mice lacking Hk1.

Circulating glucagon levels were identical in Ctrl and *αGckKO* mice in fasted conditions and similarly reduced after a short period of refeeding. This indicates that suppression of glucagon secretion in vivo relies on multiple mechanisms in addition to the *Gck*-dependent inhibitory effect. A paracrine action of insulin and somatostatin may play a role in this refed condition. Regardless of the mechanism(s) involved, it is clear that hyperglucagonemia in the fed state of *αGckKO* mice has a significant impact on hepatic glucose metabolism, as revealed by the increases in p-CREB levels, in *Pepck* and *G6pase* expression and in pyruvate-stimulated gluconeogenesis. In young mice, these defects are not associated with fed hyperglycemia or glucose intolerance because of the measured increase in glucose uptake by muscles. This increased uptake cannot be linked to higher insulin sensitivity as measured in insulin tolerance tests; it could perhaps be explained by the recently identified muscle-specific glucose sensing mechanism that increases muscle glucose uptake^20^. In adult mice, fed hyperglucagonemia is even more marked than in young mice and is associated with small glucose intolerance, and an increase in plasma insulin measured 30 min after i.p. glucose injection. Interestingly, glucose-induced insulin secretion was markedly higher in islets isolated from *αGckKO* mice than from Ctrl mice, despite similar insulin contents. This indicates that glucagon oversecretion is compensated by an adaptation of the β-cell insulin secretion capacity, which develops over time since insulin secretion by islets from young Ctrl and *αGckKO* mice was identical. This β-cell adaptation may result from direct α-cell to β-cell communication, other than through glucagon or GLP-1 signaling, or may be indirect, following changes in hepatic glucose metabolism that could increase β-cell glucose competence as described, for instance, in mice with liver-specific *Glut2* inactivation^21^.

Both type 1 and type 2 diabetes are associated with increased glucagon secretion and exacerbates the hyperglycemia resulting from the lack of insulin^22,23^. Hyperglucagonemia in type 1 diabetes may be caused by the total loss of insulin secretion, and of its inhibitory effect on α-cells. In type 2 diabetes, oral glucose fails to normally suppress glucagon secretion^24^, and insulin resistance of the α-cells may explain part of this defect^25^. Our present findings suggest that defects in Gck expression and/or in the regulation of its enzymatic activity, which can be controlled by various intracellular regulators^26^, may participate in the unrestrained secretion of glucagon in diabetes. As mentioned above^14^, plasma glucagon levels are suppressed at a higher glycemic levels in MODY2 patients as compared to control individuals. Our data suggest that this deregulation can be explained, at least in part, by reduced Gck activity in α-cells. Finally, our findings also suggest that *Gck* activators, which are being evaluated in the therapy of diabetic hyperglycemia^27^, work not only by increasing insulin secretion and hepatic glucose uptake, but also by reducing glucagon secretion by increasing glycolytic activity and the ATP/ADP ratio to block K_ATP_ channels.

Collectively, our data demonstrate the role of Gck in the glucose-dependent suppression of glucagon secretion, and identify a glucose-signaling step in α-cells whose defect can contribute to disturbances of glucose homeostasis, principally through deregulation of hepatic glucose metabolism. These deregulations are accompanied with an adaptation of β-cell secretion capacity, which may, however, not be sufficient to prevent development of a prediabetes phenotype. Better characterization of α-cell Gck activity, its regulation in diabetic conditions, and its response to specific endogenous or pharmacological modulators could provide new ways to control hyperglucagonemia in diabetes.

## Methods

### Animals

Male and female mice of a C57B6/J background were housed in groups (from 2 to 5 mice per cage), and maintained at 23 °C on a 12 h light/dark cycle with ad libitum access to chow diet (Diet 3436; Provimi Kliba AG, Kaiseraugst, Switzerland). Studies were conducted in animals of 18–36 weeks of age, and included age-matched and sex-matched littermate control mice. For all experiments, the mice were randomly assigned to experimental groups to ensure an unbiased distribution of animals. No blinding was used. All animal procedures were performed at the University of Lausanne and were reviewed and approved by the Veterinary Office of Canton de Vaud. The numbers of animals studied per genotype are indicated within each experiment.

### Generation of *αGckKO* mice

Gcg-cre mice^15^ were mated with Gck-floxed mice^9^ to generate Gck^lox/lox^ (Ctrl) and Gcg-Cre::Gck^lox/lox^ (*αGckKO*) mice. For some experiments, Rosa26tdtomato mice were bred with Gck^lox/lox^ mice to generate Gcg-Cre;Rosa26-tdtomato;Gck^+/+^ (Ctrl*-*Rosa26tdtomato) and Gcg-Cre;Rosa-26tdtomato;Gck^lox/lox^ mice (*αGckKO-Rosa26tdtomato*). To validate proper gene targeting, genomic DNA has been extracted from liver, hindbrain, ileum, and pancreatic islets using a Quick gDNA mini-prep kit (Zymo Research, USA). RT-PCR analysis was performed using a Biometra T3000 Thermocycler. Recombination efficiency was assessed in *αGckKO-Rosa26tdtomato* mice. Pancreata from 5-week-old mice were collected, fixed with 4% paraformaldehyde, and embedded in paraffin. Sections that were 5-μm-thick were stained with guinea pig anti-glucagon (Linco, diluted 1:500). Recombination efficiency was calculated as the percentage of glucagon-positive cells that also expressed tdtomato.

### Histomorphometric analysis

Pancreata were fixed with 4% paraformaldehyde and embedded in paraffin, and 5-μm-thick sections were prepared. For detection of insulin and glucagon, the sections were heated in a microwave in a citrate buffer (12 mM, pH 6.0), preincubated in a permeabilization blocking buffer (0.1 M PBS pH 7.4, 2% bovine serum albumin, 0.1% Tween 20), and incubated overnight at 4 °C with polyclonal guinea pig anti-insulin (diluted 1:400) or anti-glucagon (diluted 1:500) antibodies. The sections were then incubated for 90 min with a biotinylated goat anti–guinea pig antibody (diluted 1:750), incubated for 30 min with a biotin–avidin complex (Vector Lab), stained using DAB (Sigma-Aldrich), and weakly counterstained with Mayer’s hematoxylin (Millipore) without differentiation. α-cell masses and β-cell masses were calculated by measuring α-cell surface area or β-cell surface area using ImageJ software (http://rsbweb.nih.gov/ij/); 5–6 sections per pancreas and 3 pancreata were analyzed per genotype, representing a total of >500 islets analyzed per group. α-cell mass and β-cell mass were then calculated based on individual pancreas weight.

### Pancreatic insulin and glucagon content

Pancreata were rapidly extracted, weighed, placed in acid ethanol buffer (75% EtOH; 0.55% HCl), and minced rapidly. After overnight incubation at 4 °C, the samples were centrifuged at 1800×*g* for 20 min. Insulin and glucagon content of the supernatant was then assessed by radioimmunoassay (Merck Millipore), using insulin and glucagon standards, and expressed relative to initial pancreatic weight.

### Pancreatic islet isolation

Before removal of the pancreas, a solution of Liberase TL 0.1 mg/mL (Roche) in quenching buffer (Hank’s buffer + 25 mM HEPES) was injected into the pancreatic duct (2 mL/mouse). The pancreas was incubated at 37 °C for 14 min, after which the digestion was stopped by adding cold quenching buffer supplemented with 0.2% BSA, followed by 10 seconds of vigorous shaking, and 2 min centrifugation at 1200×*g*. The pellet was then washed twice, resuspended in 20 mL of cold RPMI (containing 11.1 mM glucose, 10% FBS, 1mM L-glutamine, and 1% Pen/strep), and filtered through a 400-μm diameter wire mesh to remove the undigested pancreas. The solution was then filtered through an inverted 100-μm wire mesh cell strainer, and the remaining islets were released by flipping the cell strainer in a 10 cm Petri dish containing RPMI. The islets were allowed to recover at least 16 h before any experimental procedure.

### Hormone release measurements

Measurements of insulin and glucagon secretion were performed using the static incubations of islets isolated from 18-week-old mice. The islets were pre-incubated for 30 min at 37 °C in Krebs-Ringer bicarbonate buffer (KRB), pH 7.4, supplemented with 10 mM Hepes, 0.1% bovine serum albumin, and 1 mM glucose. Each incubation vial was gassed with 95% O_2_ and 5% CO_2_ to obtain constant pH and oxygenation. After pre-incubation the buffer was changed to a medium containing the different conditions to be tested (1, 6, 20 mM glucose, and 1 mM glucose + 200 µM tolbutamide). Twelve islets per milliliter medium were incubated for 60 min. All incubations were performed at 37 °C in a metabolic shaker (30 cycles per minute). Immediately after incubation, the aliquots of the medium were removed for an in-house assay of insulin and glucagon^28^.

Measurements of insulin secretion were also performed on islets isolated from 36-week-old mice. Batches of 10 islets/milliliter were pre-incubated for 2 h at 37 °C in KRB supplemented with 2.8 mM glucose. Insulin secretion was assessed in the supernatant after 60 min incubation in the presence of 2.8, 10, or 20 mM glucose. At the end of each static incubation, the islets were collected and lysed in acid ethanol to assess insulin and glucagon content.

### ATP imaging

Intact islets isolated from randomly chosen Ctrl and *αGckKO-Rosa26tdtomato* mice were infected with the adenovirus encoding the ATP/ADP sensor Perceval^16^ at ~10^6^ PFU/islet. Imaging experiments were performed on a Zeiss 510 META upright confocal microscope, using a 40 × (n.a. 0.8) objective. The islets were perfused with extracellular solution containing (in mM): 140 NaCl, 3.6 KCl, 0.5 MgSO_4_, 1.3 CaCl_2_, 0.5 NaH_2_PO_4_, 5 NaHCO3, and 5 HEPES (pH 7.4 with NaOH). Glucose, methyl-succinate, and FCCP have been added as indicated in Fig. 1i and Supplementary Figs. 2, 3. Excitation/emission wavelengths were 490/535 nm. Images were acquired at a frequency of 0.05–0.1 Hz. Imaging data were background-subtracted, analyzed, and presented as the increase in F/F_0_ using ImageJ and Igor Pro software (Wavemetrics).

### Electrophysiology

All electrophysiological measurements were carried out using an EPC-10 amplifier (Heka Elektronik, Lambrecht/Pfalz, Germany) and Pulse (version 8.80) software. Pancreatic islets were cultured overnight and transferred into a recording chamber mounted in an upright microscope (Nikon Eclipse E600FN, Japan), where islets were perfused with physiological saline heated by an inline heater (chamber temperature was kept at 32–34 °C). Electrical activity, transmembrane currents, and cell capacitance were recorded from randomly chosen cells on the peripheral of the islets. α-cells were identified by the expression of fluorescent protein tdtomato (see *Mouse Validation*). α-cells were identified by their electrical activity in response to glucose and lack of tdtomato fluorescence.

Electrical activity and K_ATP_ conductance were recorded using perforated patch-clamping technique. Perforating reagent gramicidin (0.4 mg/L) was added into intracellular solution containing (in mM): 76 K_2_SO_4_, 10 KCl, 1 MgCl_2_, and 5 HEPES (pH 7.35 with KOH). Extracellular solution contains (in mM): 140 NaCl, 3.6 KCl, 0.5 MgSO_4_, 1.5 CaCl_2_, 0.5 NaH_2_PO_4_, 5 NaHCO3, and 10 HEPES (pH 7.4 with NaOH). Tolbutamide and/or D-glucose were added at concentrations as indicated. After the experiments, the membrane potential recordings were exported as ASCII files and converted to ABF files (axon binary file) using ABF utility software (version 2.1.57, Synaptosoft Inc, NJ, USA). The resultant ABF files were then imported into Clampfit software (version 9.2.0.11, Molecular Devices, CA, USA) for analysis. Action potentials were detected and parameters (peak potential, antipeak potential, and firing frequency) were extracted using the ‘template search’ function of Clampfit.

Depolarization-triggered cell exocytosis was monitored as increase in membrane capacitance. This was detected by the Lindau–Neher technique using ‘Sine + DC’ feature of the lock-in module of the Pulse software. The sine wave used was 20 mV of amplitude and 1.25 kHz of frequency. The intracellular solution used for capacitance measurement contains (in mM): 125 Cs-glutamate, 10 CsCl, 10 NaCl, 1 MgCl_2_, 5 HEPES, 0.05 EGTA, and 3 ATP (pH 7.2 with CsOH). The extracellular solution contains (in mM): 118 NaCl, 5.6 KCl, 1.2 MgCl_2_, 2.6 CaCl_2_, 5 HEPES, 20 tetraethylammonium Cl (TEA-Cl), and 1 D-glucose (pH 7.4 with NaOH).

Recording electrodes were pulled from borosilicate glass (Harvard Apparatus, MA, USA) and had resistance of ~7 MΩ when filled with intracellular solutions.

### Glucose and pyruvate tolerance tests

The animals were fasted for 15 h, then independently housed, and i.p. injected with a bolus of glucose (2 g/kg b.w.). For pyruvate tolerance test, the mice in a random-fed state (within the first 60 min of light phase) received an i.p. injection of sodium pyruvate (2 g/kg b.w., Sigma Aldrich). Blood glucose was monitored for 120 min using a glucometer (Breeze2, Bayer) by tail vein sampling.

### In vivo biochemical measurements

Plasma glucagon levels were quantitated by radioimmunoassay (Merck Millipore) and by ELISA (Mercodia). Plasma insulin levels were assessed by ultra-sensitive ELISA (Mercodia).

### Western blotting analysis

A portion of mouse liver were homogenized in ice-cold homogeneisation buffer (in mM: 300 sucrose, 10 HEPES pH 7.9, 10 KCl, 1.5 MgCl2, 0.1 EGTA, and 0.5 PMSF), supplemented with 1 mM sodium orthovanadate, protease, and phosphatase inhibitor cocktail tablets (Roche). Proteins from nuclear fractions were extracted, and the protein content was determined by bicinchoninic acid assay (Pierce, Thermo Scientific). Equal amounts of protein (30 μg) were loaded onto 10% polyacrylamide gels and electrophoresed. Transfer to nitrocellulose membranes was performed using the Mini Trans-Blot apparatus from Bio-Rad. The membranes were blocked in 5% BSA (w/v) in Tris-buffered saline with Tween-20 (TBST) (in mM: 15 Tris-HCl, 137 NaCl, 0.1% Tween-20, pH 7.6) at room temperature (RT), then incubated with anti-CREB (Cell Signaling Technology) and anti-phospho-CREB (Ser-133) (Cell Signaling Technology) antibodies (diluted 1:1000) in 5% BSA/TBST overnight at 4 °C. After TBST 3 × 5 min washes, the membranes were incubated for 1 h at RT with secondary anti-rabbit HRP-conjugated antibody (GE Healthcare) diluted 1:8000 in 5% BSA/TBST, followed by further washes. Bands corresponding to the specific proteins were visualized using enhanced chemiluminescence reagent (Advansta). Digital images were acquired with Fusion FX7 system (Vilber Lourmat) and Bio-1D software (Vilber Lourmat) for quantification and normalization. The same membranes were reprobed with anti-β-actin antibodies to confirm the equal loading of proteins for each sample.

### Liver RNA extraction and quantitative PCR analysis

Total RNA was prepared from livers using peqGOLD TriFast according to the manufacturer’s instructions (PeqLab). First-strand cDNA was synthesized from 1 μg of total RNA using random primers (Promega) and M-MLV reverse transcriptase (Promega). Real-time PCR was performed using Power SYBR Green Master Mix (Applied Biosystems). All reactions were normalized to β-actin levels. Specific mouse primers for each gene are listed in Supplementary Table 1.

### Radioactive 2-deoxy-glucose (2-DG) uptake assay

The animals were processed in the morning in the random-fed state. The mice received a bolus of ^14^C-2-deoxy-D-glucose (Perkin-Elmer; dil. 10uCi in 100 ul saline) by retro-orbital injection under anesthesia with isoflurane using an anesthetic mask on a heating pad. The anesthesia was maintained for 2 min after the injection. The mice were then placed in cages without water or food. Blood glucose was measured (Breeze2, Bayer) and blood samples (30 ul) were collected from the tail tip at 0, 5, 15, 25, 35, and 45 min post-injection. After the last blood sampling, the mice were killed by cervical dislocation under isoflurane anesthesia. Tissues were immediately dissected and frozen for further assessment of ^14^C-2-deoxy-D-glucose-6-phosphate (2-DG-6-P) content. The Plasma radioactivity was determined at each time point by liquid scintillation counting, in order to calculate the area under the curve of the plasma tracer decay. For the determination of tissue 2-DG-6-P content, the tissue samples were homogenized, and the supernatants were passed through ion-exchange columns to separate 2-DG-6-P from 2-DG. Tissue 2DG uptake was calculated by normalizing the tissue 2DG-6P content (as disintegrations per minute) to the tissue weight and to the AUC of the plasma tracer decay.

### Data representation and statistics

All collected data were included without data exclusion. Statistical analysis was performed using GraphPad Prism 5.0c either by Student’s *t-*test, One-way, and Two-way ANOVA followed by Bonferroni post hoc test. The data distribution was assumed to be normal. Data are expressed as mean ± SEM. *p*-values less than 0.05 were considered significant. Other statistical methods were mentioned and indicated where they were used. No statistical methods were used to pre-determine sample sizes, but sample sizes are similar to those used in our previous studies.

### Data availability

The data that support the findings of this study are available from the corresponding author upon reasonable request.

## Electronic supplementary material

Supplementary Information

Peer Review File
