# Supplementary material for: α-cell glucokinase suppresses glucose-regulated glucagon secretion
Source: Nat Commun. 2018 Feb 7;9:546. doi: 10.1038/s41467-018-03034-0 (PMC5803227; doi:10.1038/s41467-018-03034-0)
Supplement: Supplementary file 1 — Supplementary Information [file 41467_2018_3034_MOESM1_ESM.pdf]

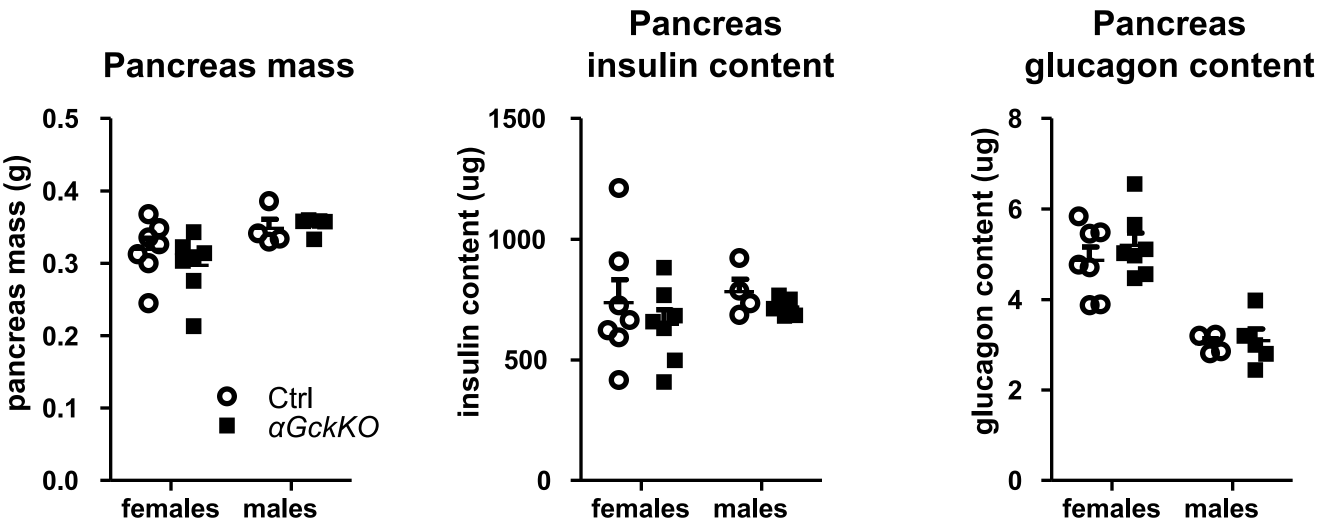


Supplementary Figure 1. Pancreas mass, pancreatic insulin and glucagon content in 18 week-old Ctrl and *αGckKO* male and female mice. (related to figure 1). N=5 males and n=7 females per genotype.

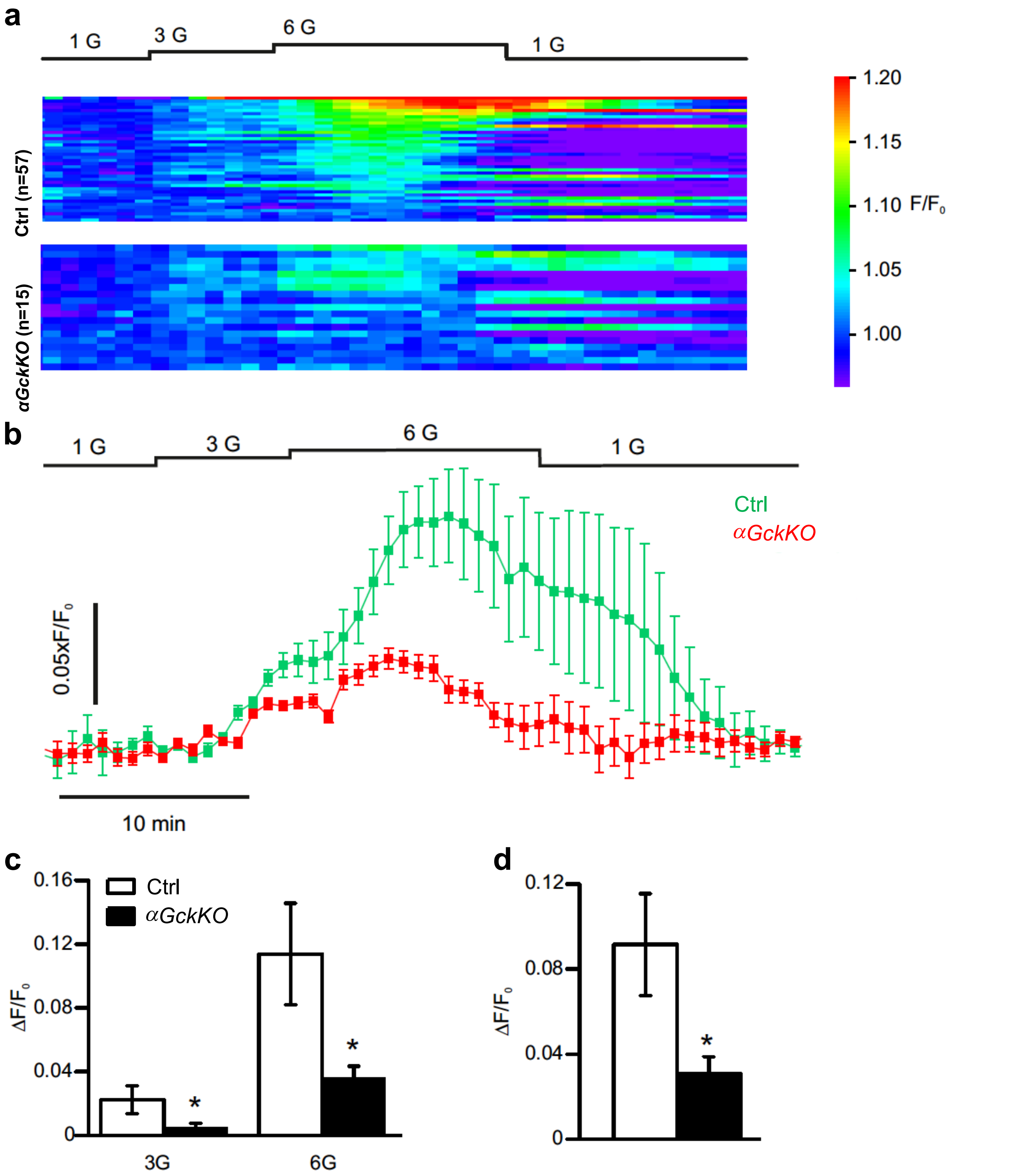


Supplementary figure 2. ATP/ADP ratio in α-cells from 18-20 week-old Ctrl and *αGckKO* female mice (related to figure 1). **a)** Heat map of Perceval fluorescence in α-cells in the presence of the indicated glucose concentrations (mM). n=57 for Ctrl and n=15 for *αGckKO*. **b)** Summary of Perceval fluorescence changes in Ctrl and *αGckKO* α-cells. n=57 for Ctrl and n=15 for *αGckKO*. **c)** Perceval fluorescence in Ctrl and *αGckKO* α-cells when glucose was elevated from 1 mM to 3 or 6mM. *p<0.05; Student *t*-test. **d)** Perceval fluorescence in Ctrl and *αGckKO* α-cells when glucose was elevated from 3 to 6 mM. *p<0.05; Student *t*-test. Data are represented as mean ±s.e.m.

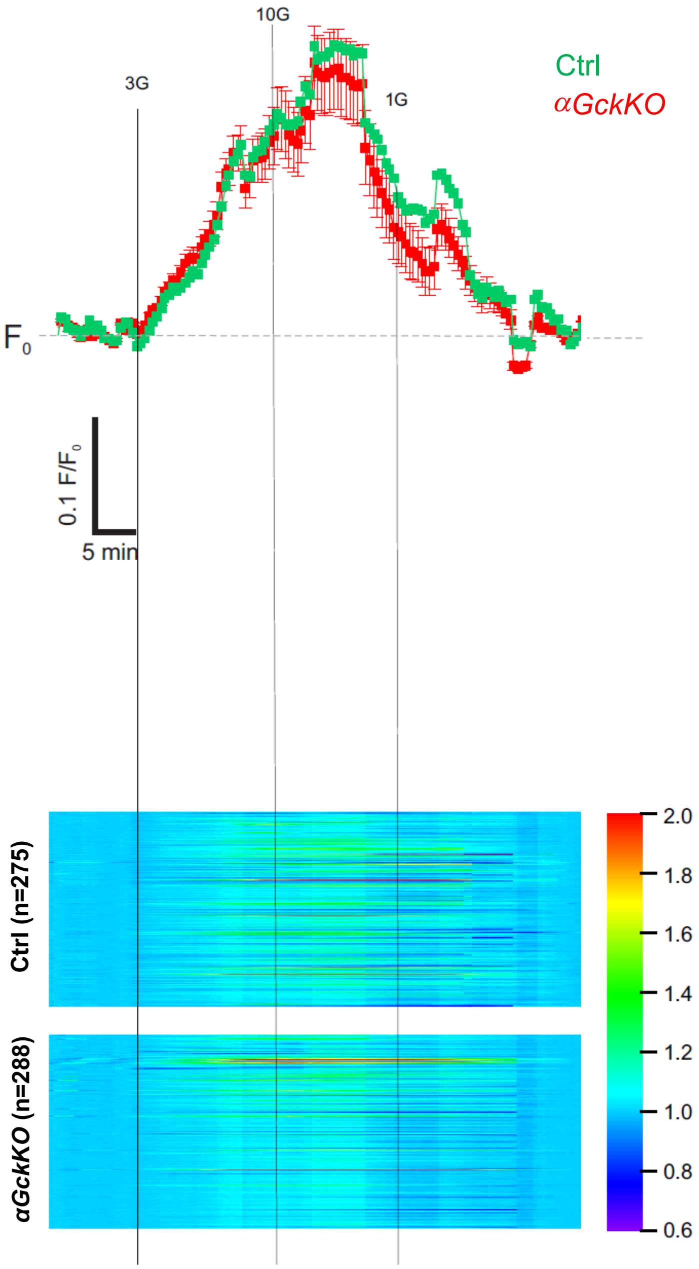


Supplementary figure 3. ATP/ADP ratio in β-cells from 18-20 week-old Ctrl and *αGckKO* female mice (related to figure 1). Heat map of Perceval fluorescence in β-cells in the presence of the indicated glucose concentrations (mM). n=275 for Ctrl and n=288 for *αGckKO*. Data are represented as mean ±s.e.m.

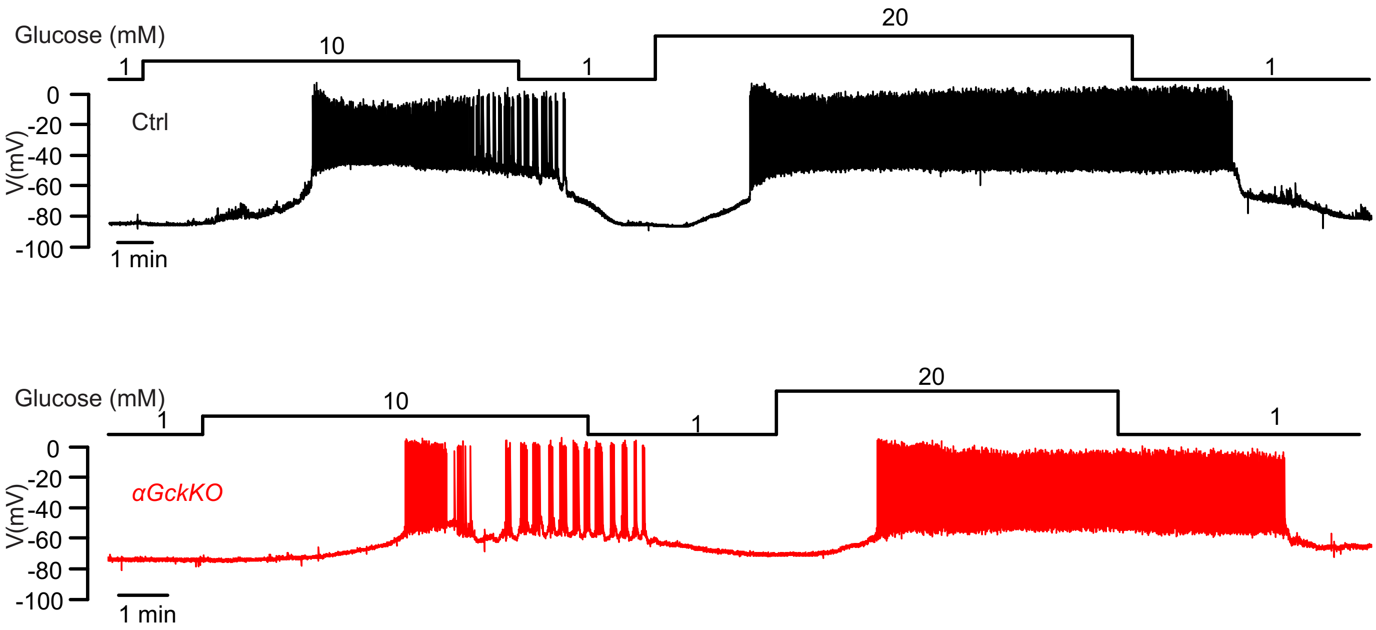


Supplementary figure 4. Example of β-cell electrical activity measured in whole islets from 18 week-old Ctrl (black, n=4) and *αGckKO* (red, n=4) female mice (related to figure 2).

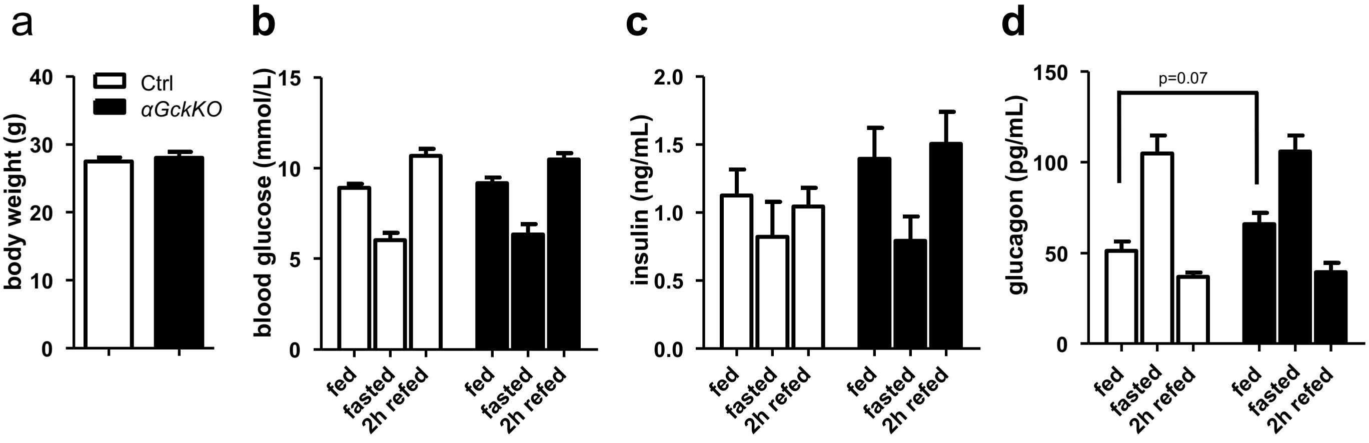


Supplementary figure 5. Metabolic profile of 20 week-old Ctrl and *αGckKO* male mice (related to figure 3). (**a**) Body weight, (**b**) Blood glucose, (**c**) Plasma insulin, and (**d**) Plasma glucagon. N=11 Ctrl and n=11 *αGckKO* mice. Student's t-test. Data are represented as mean ±s.e.m.

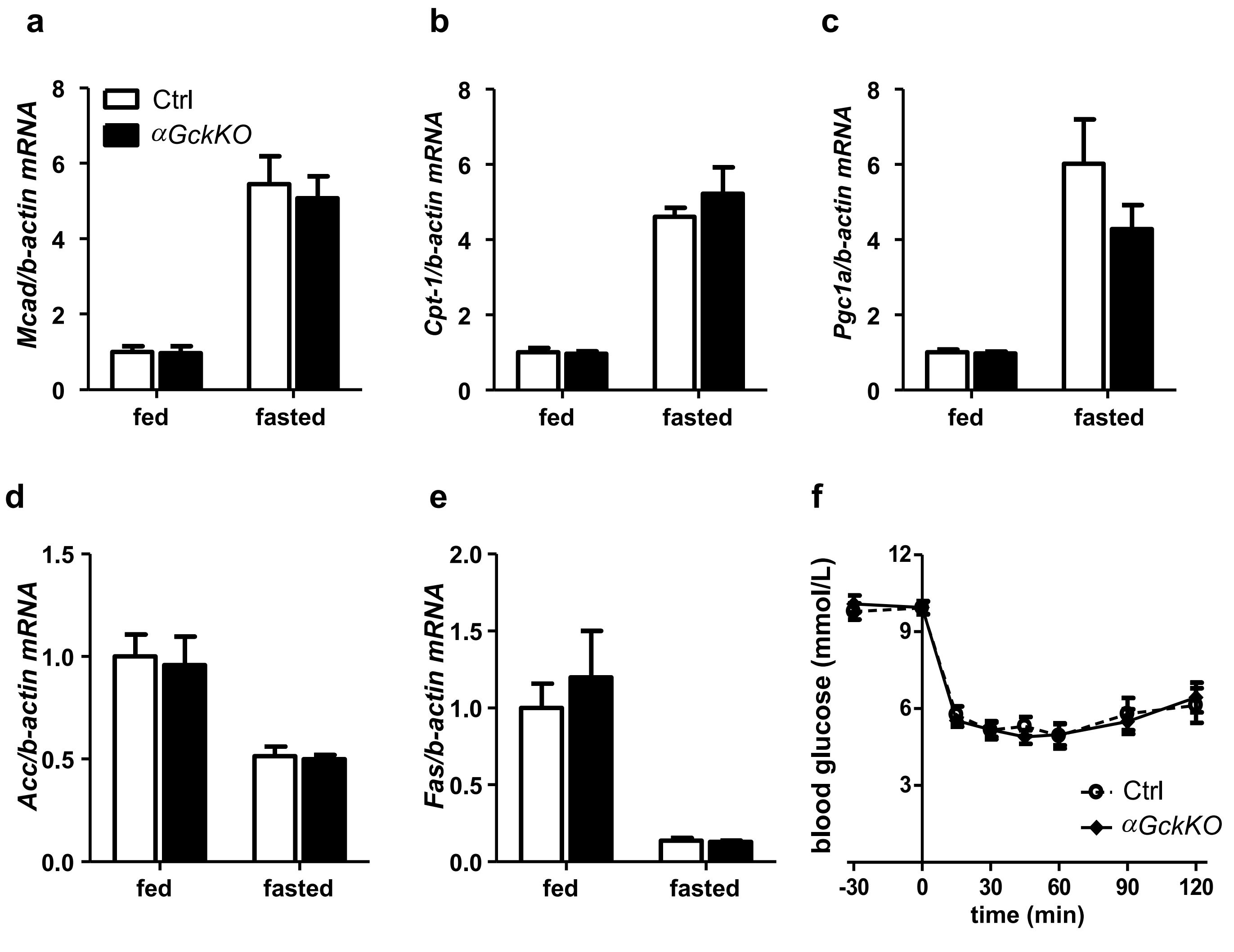


Supplementary Figure 6. (**a-c**) Fatty acid oxidation (*Mcad, Cpt1, Pgc1a*) and (**d,e**) synthesis (*Acc, Fas*) gene expression in the liver of 18 week-old Ctrl and *αGckKO* analyzed by qRT-PCR (related to figure 3). **f**) Intraperitoneal insulin tolerance test in 18 week-old female mice. 6 hrs fasted mice were injected with 0.7 U/kg i.p. insulin. N=9 Ctrl and 11 *aGckKo* mice. Data are represented as mean ±s.e.m.

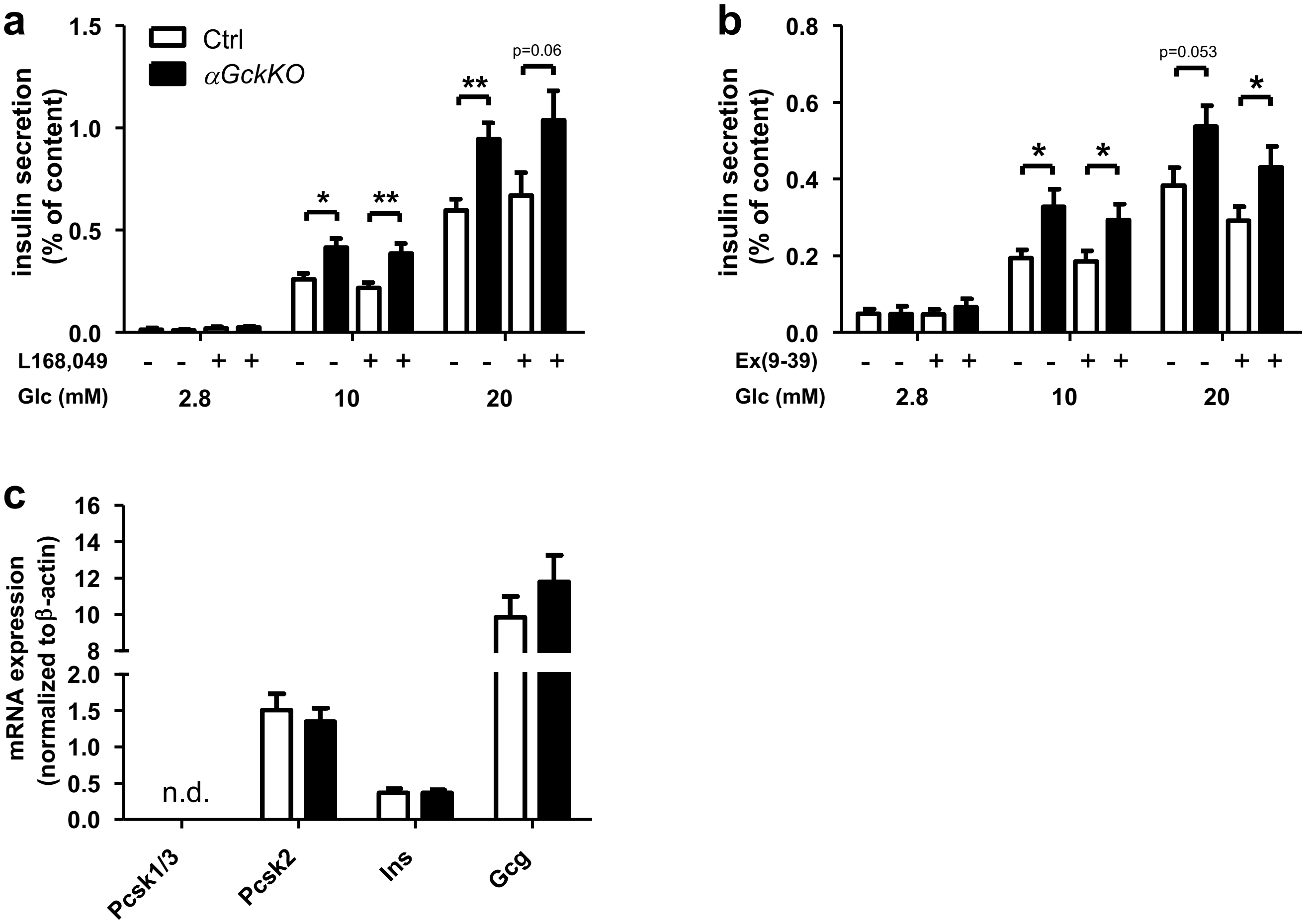


Supplementary Figure 7. Increased glucose-stimulated insulin secretion by *αGckKO* mouse islets is not reduced by glucagon and GLP-1 antagonism (related to figure 4). **a)** Glucose stimulated insulin secretion in islets isolated from 36 week-old female mice in presence of the GcgR antagonist, L168,049 100nM. **b)** Same as **(a)** but in presence of the Glp1R antagonist, Ex(9-39) 100nM. **c)** *Pcsk1/3, Pcsk2, Ins1, and Gcg* expression levels in FACS-purified α-cells from 36 week-old female mice. Data represent the average of four independent experiments, each performed in duplicate. *p<0.05; **p<0.01; Student *t*-test. Data are represented as mean ± s.e.m.

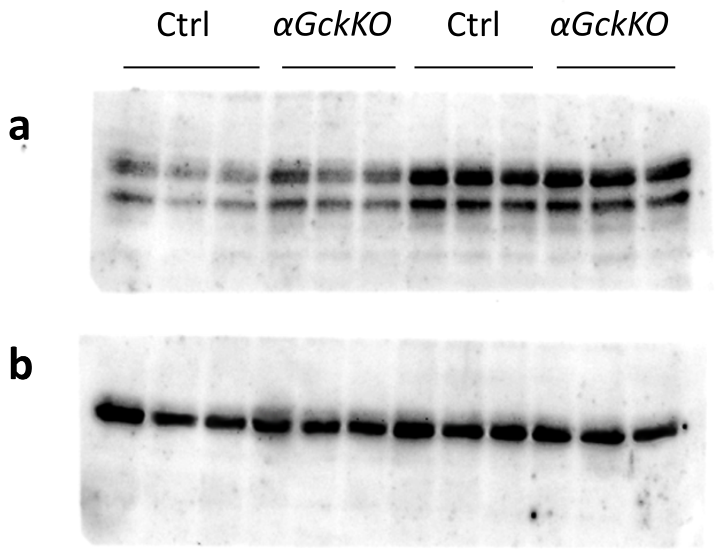


Supplementary Figure 8. Uncropped images of pCREB and CREB immunoblotting in liver lysates. **a)** Uncropped image of immunoblotting for pCREB (43 kDa - upper image) and **(b)** CREB (43 kDa - lower image) in liver samples in random fed state and under ON fasting from 18 week-old Ctrl and *αGckKO* female mice (related to figure 3).

Supplementary Table 1. Primer sequences for genotyping (Gck) and for q-RT-PCR analysis.

| Primer | Forward 5'-3' | Reverse 5'-3' |
|---|---|---|
| Gck (genotyping) | GTTCAGGTTATAGTACATCACAGCAG | ATTTGCCTTGTCAACTTGTCC |
| Gck | ACTGCGGAGATGCTCTTTGA | TCTCGGAGAAGTCCCACGAT |
| G6Pase | TCCTGGGACAGACACACAAG | CAACTTTAATATACGCTATTGG |
| Pepck | CTTCTCTGCCAAGGTCATCC | TTTTGGGGATGGGCAC |
| Mcad | GCTCGTGAGCACATTGAAAA | CATTGTCCAAAAGCCAAACC |
| Cpt-1 | ATCATGTATCGCCGCAAACT | CCATCTGGTAGGAGCACATGG |
| Pgc1α | AGACAAATGTGCTTCCAAAAAGAA | GAAGAGATAAAGTTGTTGGTTTGGC |
| Acc | ATTGGGCACCCCAGAGCTA | CCCGCTCCTTCAACTTGCT |
| Fas | CGTATATGTGAACAGCGC | AGGTCTCGGATGCCTA |
| β-actin | GCTTCTTTGCAGCTCCTTCGT | ATATCGTCATCCATGCGCAAC |
| Pcsk 1/3 | ACCAGGTGCTGCATATCTCG | CACAATGACTGCACGGAGAC |
| Pcsk2 | TTTCGGTCAAATCCTTCCTG | TGCAAAGGCCAAGCGAAGAC |
| Ins1 | CCATCAGCAAGCAGGTCATTGTT | AACGCCAAGGTCTGAAGGTCC |
| Gcg | CCAAGATTTTGTGCAGTGGTT | GGTAAAGGTCCCTTCAGCAT |
