# Supplementary material for: α-cell glucokinase suppresses glucose-regulated glucagon secretion
Source: Nat Commun. 2018 Feb 7;9:546. doi: 10.1038/s41467-018-03034-0 (PMC5803227; doi:10.1038/s41467-018-03034-0)
Supplement: Supplementary file 2 — Peer Review File [file 41467_2018_3034_MOESM2_ESM.pdf]

Reviewer #1 (Remarks to the Author):

This manuscript describes a study demonstrating the importance of GK in pancreatic alpha cells. This is an extremely important topic of investigation as need to learn more basic science about the regulation of glucagon secretion. The author use a novel mouse model system that specifically deletes the GK gene in alpha cells to demonstrate the importance of GK in the repression of glucagon secretion in low glucose concentrations. The authors demonstrate the electrophysiological consequences of GK deletion and the physiological consequences by performing a thorough phenotyping of glucose homeostasis. The study is well done, well written and the data are clean and convincing. There are a few minor issues that should be addressed:

1. Care should be taken not to conflate tdTomato expression with excision.

2. There needs to be a reference or detailed description of the adenovirus expressing the ATP/ADP sensor, Percival.

3. I may be misunderstanding this, but the overshooting action potential in control cells in Fig 2a look to be closer to 20 mV at 1 mM glucose. The figure should be more representative, or this passage should be clarified.

Reviewer #2 (Remarks to the Author):

Studies have shown that the glucose sensing enzyme glucokinase is expressed in the pancreatic alpha cell and that glucose causes direct inhibition of glucagon secretion. Basco and colleagues now demonstrate that alpha cell glucokinase is involved in glucose-regulated glucagon secretion. To this end, they generated a mouse with alpha cell-specific inactivation of glucokinase. Overall the mouse has a mild phenotype, although females develop glucose intolerance with age. The ex-vivo studies show lack of suppression of glucagon secretion with increasing glucose concentrations, which is secondary to reduced ATP production and consequently low ratio of ATP:ADP. This ratio is an important regulator of K-ATP channel and electrical activity, and eventually glucagon secretion. This is confirmed in the present study where alpha cells which lack glucokinase do not suppress K-ATP channel and electrical activity to glucose stimulation accounting for the lack of suppression of glucagon secretion. The study is well conducted and written. I have a few major concerns with the study as outlined below:

1. Glucokinase has a Km for glucose around 8 mM. Previous work from the authors have shown complete suppression of glucagon secretion at glucose concentration below 5 mM. How do the authors explain the apparent complete disconnect between the glucose requirements for glucokinase and suppression of glucagon secretion? This is also an important question since glucokinase controls glucose-induced insulin secretion which is only increased at glucose levels that causes complete suppression of glucagon release. I encourage the authors to conduct studies with additional glucose concentrations to establish dose-response relationships between glucokinase-mediated ATP production and glucagon secretion to help explain this discrepancy.

2. It’s surprising that mice with alpha cell-specific inactivation of glucokinase show higher glucose uptake in skeletal muscle. This is an important observation and mechanism should be established.

3. The authors should address why female mice that lack glucokinase develop mild glucose intolerance over time. 36 week old mice are not “old” as stated in certain places in the manuscript. They are adult.

4. The authors state in the discussion that activators of glucokinase suppress glucagon secretion. Are there experimental evidence for this? If so it should be cited.

Reviewer #3 (Remarks to the Author):

This is an interesting paper wherein the authors report the inactivation of glucokinase in alpha cells in a mouse model. The ensuing phenotype argues for the relevance of glucokinase in the regulation of glucagon secretion. While a majority of the phenotype is accounted for by the experiments there are some issues that are puzzling and require some additional work. These effects follow a consistent pattern and are related to the secretion of insulin from the beta cells; thus, clearly indicating that interfering with alpha cell glucokinase expression impacts function of neighboring beta cells. Some of these issues are listed below:

1. The insulin secretion in response to Tolbutamide (Fig 1) is very muted. One would have anticipated a robust secretory effect. How do the authors explain this? What happens to insulin and glucagon response to L-arginine stimulation? These data should be included.

2. In most insulin "secretion" data C-peptide should be reported to avoid confounding issues regarding insulin resistance effects - especially since hepatic function is likely altered.

3. The enhanced secretion of insulin in older mice is intriguing and suggests potential functional interplay between alpha and beta cells. The authors don't have a robust explanation for this beta cell adaptation. They mention that this finding could reflect an altered beta cell glucose competence either due to a paracrine effect between alpha and beta cells or an indirect effect via altered hepatic metabolism similar to what is seen in mice with hepatic inactivation of glut2. However, I am sure the authors will agree that additional experimentation is necessary to clarify this rather intriguing effect.

4. I wonder if the word "critical" is really appropriate in the title.

Reviewer #4 (Remarks to the Author):

This study shows the importance of glucokinase in glucose-regulated glucagon secretion. The question is important, the experiments are convincing and the data are well presented. While the results clearly show the mechanistic importance of glucokinase in the regulation of glucagon, I would have been interested to learn a little more on the broader significance of this finding: how does glucokinase inactivation impact on other modulators of alpha cell function such as amino acids and GLP-1 at different glucose concentrations? Does glucokinase impact on processing and secretion of alpha cell derived GLP-1? Does glucokinase deficiency impact on the influence of a diabetic milieu (high fat diet in vivo, and glucose, fatty acids and cytokines in vitro) on alpha cell function? While I do not consider these experiments as mandatory, some would make the paper more attractive.

Due to the quality of the manuscript, I have only two specific minor comments:
1. Abstract: „…over time, induces a pre-diabetic phenotype”. The wording “pre-diabetic” is not completely accurate: because the precise role alpha cell glucokinase in diabetes is not elucidated, it remains to be shown whether observed changes would really contribute to a diabetic phenotype.
2. Typo: Discussion Line 186: glucose intolerance (not tolerance).

We would like to thank the reviewers for their positive and constructive assessment of our manuscript. We have now prepared detailed answers and additional experiments to address all of their concerns, as described below.

Reviewer #1 (Remarks to the Author):

This manuscript describes a study demonstrating the importance of GK in pancreatic alpha cells. This is an extremely important topic of investigation as need to learn more basic science about the regulation of glucagon secretion. The author use a novel mouse model system that specifically deletes the GK gene in alpha cells to demonstrate the importance of GK in the repression of glucagon secretion in low glucose concentrations. The authors demonstrate the electrophysiological consequences of GK deletion and the physiological consequences by performing a thorough phenotyping of glucose homeostasis. The study is well done, well written and the data are clean and convincing. There are a few minor issues that should be addressed:

We thank the reviewer for his/her very positive comments.

1. Care should be taken not to conflate tdTomato expression with excision.

We agree that tdTomato expression is not a direct measure of recombination of the *Gck*^{lox} alleles. Accordingly, we have modified the second sentence of the RESULTS paragraph to: "These mice were further crossed with *Rosa26-tdtomato* mice and approximately 70% of the glucagon-positive cells also expressed tdtomato (Figure 1A), indicating that a large majority of α-cells express the Cre recombinase".

2. There needs to be a reference or detailed description of the adenovirus expressing the ATP/ADP sensor, Perceval.

The following reference has now been added (Results, page 6, 5th line and Methods, page 17, first sentence of third paragraph): Berg et al., A genetically encoded fluorescent reporter of ATP:ADP ratio. Nature Methods 2009 (Ref 17).

3. I may be misunderstanding this, but the overshooting action potential in control cells in Fig 2a look to be closer to 20 mV at 1 mM glucose. The figure should be more representative, or this passage should be clarified.

A new Fig 2a has been prepared that is more representative. Inclusion of additional electrophysiological data is reflected in small changes in the quantitative analysis of Fig. 2b.

Reviewer #2 (Remarks to the Author):

Studies have shown that the glucose sensing enzyme glucokinase is expressed in the pancreatic alpha cell and that glucose causes direct inhibition of glucagon secretion. Basco and colleagues now

demonstrate that alpha cell glucokinase is involved in glucose-regulated glucagon secretion. To this end, they generated a mouse with alpha cell-specific inactivation of glucokinase. Overall the mouse has a mild phenotype, although females develop glucose intolerance with age. The ex-vivo studies show lack of suppression of glucagon secretion with increasing glucose concentrations, which is secondary to reduced ATP production and consequently low ratio of ATP:ADP. This ratio is an important regulator of K-ATP channel and electrical activity, and eventually glucagon secretion. This is confirmed in the present study where alpha cells which lack glucokinase do not suppress K-ATP channel and electrical activity to glucose stimulation accounting for the lack of suppression of glucagon secretion. The study is well conducted and written. I have a few major concerns with the study as outlined below:

1. Glucokinase has a Km for glucose around 8 mM. Previous work from the authors have shown complete suppression of glucagon secretion at glucose concentration below 5 mM. How do the authors explain the apparent complete disconnect between the glucose requirements for glucokinase and suppression of glucagon secretion? This is also an important question since glucokinase controls glucose-induced insulin secretion which is only increased at glucose levels that causes complete suppression of glucagon release. I encourage the authors to conduct studies with additional glucose concentrations to establish dose-response relationships between glucokinase-mediated ATP production and glucagon secretion to help explain this discrepancy.

We have previously shown that 6 mM glucose increases the intracellular ATP/ADP ratio (Zhang et al., Cell Metab. 2013, ref 3). In unpublished experiments, we have shown that the effect of 6mM glucose is ~50% of that seen at 17 mM glucose. Nevertheless, glucagon secretion is maximally inhibited at 5-6 mM glucose (and as strongly as that produced by tolbutamide) (Walker JN et al., 2011, ref 19) and higher glucose concentrations are – if anything and paradoxically – less inhibitory. Thus, the increase in ATP produced by 6mM glucose is sufficient to fully inhibit K_{ATP} channel activity in the alpha-cell. This is similar to what is seen in beta cell where K_{ATP} channel activity is also inhibited at glucose concentrations much lower than those triggering insulin secretion and increasing intracellular ATP, which is a consequence of the KATP channel's high ATP sensitivity.
To experimentally address this comment, we have performed a new experiment, whose results are presented in Fig S2 and which shows that 3 mM glucose already significantly increases the ATP/ADP ratio in Ctrl α cells but not in α cells from *αGckKO* mice. This mentioned in the Results , page 6, line 12.

2. It's surprising that mice with alpha cell-specific inactivation of glucokinase show higher glucose uptake in skeletal muscle. This is an important observation and mechanism should be established.

We did not observe increased insulin sensitivity in *αGckKO* mice, as assessed by insulin tolerance tests (new Fig S6f), and as shown by identical plasma insulin levels in Ctrl and *αGckKO* mice (Figure 2). Therefore the observed redistribution of glucose to skeletal muscles is not easily explained but may be accounted for by the recently described muscle-specific glucose sensing mechanism that increases muscle glucose uptake (Meng ZX, et al., Mol Cell 2017, 66:332, new ref 21). We have added the following text to page 9, last sentence of first paragraph: "Normal glycemic control was, however, preserved because of increased glucose uptake in skeletal muscles, which was however not associated with

increased insulin sensitivity as measured by insulin tolerance test (Supplementary Fig. 6f)", and the following text in the Discussion, bottom of page 11: "This increased uptake cannot be linked to higher insulin sensitivity as measured in insulin tolerance tests; it could perhaps be explained by the recently identified muscle-specific glucose sensing mechanism that increases muscle glucose uptake[21]."

3. The authors should address why female mice that lack glucokinase develop mild glucose intolerance over time.

Development of glucose intolerance and diabetes is very much influenced by sex and sex hormones, and sex differences in susceptibility to develop diabetes have been reported for many genetic models. Thus, this observation is not surprising – even though a precise explanation for this particular mouse model cannot be provided without performing specific additional experiments, which we feel are beyond the scope of the present study.

36 week old mice are not "old" as stated in certain places in the manuscript. They are adult.

We replaced "old" mice with "adult" mice in the manuscript.

4. The authors state in the discussion that activators of glucokinase suppress glucagon secretion. Are there experimental evidence for this? If so it should be cited.

We do not state that glucokinase activators suppress glucagon secretion. The exact sentence is: "Finally, our findings also suggest that *Gck* activators, which are being evaluated in the therapy of diabetic hyperglycemia(1), work not only by increasing insulin secretion and hepatic glucose uptake, but also by reducing glucagon secretion by increasing glycolytic activity and the ATP/ADP ratio to block K_{ATP} channels."

On the other hand, as alluded to above, the effects of glucose on glucagon secretion involve both an inhibitory effect (that predominates at glucose concentrations up to ~6 mM that is superseded by a stimulatory effect at higher glucose concentrations (Rorsman et al., Diabetologia 2014, ref 20). In experiments using GKA50 (from Astrazeneca), the inhibitory effect of glucose is obscured by a left-shift of the stimulatory effect (that correlated with a similar left-shift of glucose-induced insulin secretion). We have not published these data as they pose a significant didactic problem and we are not aware of any published reports to this effect (possibly because others have encountered the same 'problem'). We are not convinced this paper is the right forum to discuss these complex data.

Reviewer #3 (Remarks to the Author):

This is an interesting paper wherein the authors report the inactivation of glucokinase in alpha cells in a

mouse model. The ensuing phenotype argues for the relevance of glucokinase in the regulation of glucagon secretion. While a majority of the phenotype is accounted for by the experiments there are some issues that are puzzling and require some additional work. These effects follow a consistent pattern and are related to the secretion of insulin from the beta cells; thus, clearly indicating that interfering with alpha cell glucokinase expression impacts function of neighboring beta cells. Some of these issues are listed below:

1. The insulin secretion in response to Tolbutamide (Fig 1) is very muted. One would have anticipated a robust secretory effect. How do the authors explain this?

The K_{ATP}-dependent channel blocker tolbutamide strongly depolarizes beta-cell membranes and promotes a rapid increase in insulin secretion. However, this is not sustained over time as we used tolbutamide in low glucose conditions (1mM Glc), which typically inhibit insulin secretion. For this reason, the values reported in Fig.1H are not as robust as expected because of a diluted effect of tolbutamide over 1h incubation.

What happens to insulin and glucagon response to L-arginine stimulation? These data should be included.

With respect, we do not see the relevance of these experiments. It is well established that this charged amino acid depolarizes the alpha- and beta-cells by its electrogenic entry. We acknowledge that testing if glucose retains an inhibitory effect on glucagon secretion triggered by arginine might shed light on the paracrine regulation of glucagon secretion but that such experiments are somewhat beyond the scope of this study which focuses on the glucokinase in the intrinsic regulation and to this end have established an alpha cell-specific glucokinase knockout mouse model.

2. In most insulin "secretion" data C-peptide should be reported to avoid confounding issues regarding insulin resistance effects - especially since hepatic function is likely altered.

With hindsight it would have been valuable to measure C-peptide as well but unfortunately these measurements are not feasible as we do not have any serum left – performing these measurements would require breeding a new lot of mice and repeating all the experiments.

3. The enhanced secretion of insulin in older mice is intriguing and suggests potential functional interplay between alpha and beta cells. The authors don't have a robust explanation for this beta cell adaptation. They mention that this finding could reflect an altered beta cell glucose competence either due to a paracrine effect between alpha and beta cells or an indirect effect via altered hepatic metabolism similar to what is seen in mice with hepatic inactivation of glut2. However, I am sure the authors will agree that additional experimentation is necessary to clarify this rather intriguing effect.
To answer this question we have performed new experiments with islets isolated from 36 week-old mice. We tested whether the observed increase in insulin secretion could be due to increased paracrine glucagon or GLP-1 secretion. We present a new supplementary figure (Supplementary Fig. 7) which

shows that glucose-stimulated insulin secretion is not reduced when islets are incubated in the presence of the glucagon receptor antagonist L-168,049 (Supplementary Fig. 7a) or with the GLP-1R antagonist exendin(9-39) (Supplementary Fig. 7a). We also show that there is no overexpression of *Pcsk1/3*, which encode the proconvertase required for GLP-1 processing from proglucagon (Supplementary Fig. 7c). We can thus exclude two potential mechanisms that could link alpha cell secretion activity to increased beta-cell secretion.

We have, thus, added the following text at the end of page 9 – top of page 10: "To determine whether this could be due to increased glucagon secretion from alpha cells or to increased processing of preproglucagon into, and secretion of GLP-1 from alpha cells, we performed insulin secretion experiments in the presence of the glucagon receptor antagonist L-168,049 and of the GLP-1R antagonist exendin(9-39). No inhibitory effects on glucose stimulated insulin secretion by islets from *αGckKO* mice could be observed in these conditions (Supplementary Fig. 7a,b); qRT-PCR analysis also showed that the level of *Pcsk1/3* was not increased in *αGckKO* α cells (Supplementary Fig. 7c).

We also modified the last sentence of the first paragraph of page 12 as follows (red):" This β-cell adaptation may result from direct α-cell to β-cell communication, other than through glucagon or GLP-1 signaling, or may be indirect,…"

4. I wonder if the word "critical" is really appropriate in the title.

We think the use of the word 'critical' can be justified but we are not passionate about it and would accept the somewhat less informative title: 'The role of α-cell glucokinase in glucose-regulated glucagon secretion".

Reviewer #4 (Remarks to the Author):
This study shows the importance of glucokinase in glucose-regulated glucagon secretion. The question is important, the experiments are convincing and the data are well presented. While the results clearly show the mechanistic importance of glucokinase in the regulation of glucagon, I would have been interested to learn a little more on the broader significance of this finding: how does glucokinase inactivation impact on other modulators of alpha cell function such as amino acids and GLP-1 at different glucose concentrations? Does glucokinase impact on processing and secretion of alpha cell derived GLP-1? Does glucokinase deficiency impact on the influence of a diabetic milieu (high fat diet in vivo, and glucose, fatty acids and cytokines in vitro) on alpha cell function? While I do not consider these experiments as mandatory, some would make the paper more attractive.

We are currently studying the effects of GLP-1 on glucagon secretion. The reviewer might recall that α-cell express GLP1Rs at very low levels (if at all) and we feel that this paper might not be the right place to discuss the mechanisms by which GLP-1 act on glucagon secretion (which are very interesting but better considered in a separate manuscript).

Regarding the processing of proglucagon, and also in response to question #3 of reviewer 3, we have performed additional experiments and prepared a new Supplementary Fig. 7. This shows that glucose-stimulated insulin secretion is not reduced when islets are incubated in the presence of the glucagon receptor antagonist L-168,049 (Supplementary Fig. 7a) or with the GLP-1R antagonist exendin(9-39) (Supplementary Fig. 7b). We also show that there is no overexpression of *Pcsk1/3*, which encodes the proconvertase required for GLP-1 processing from proglucagon (Supplementary Fig. 7c). We can thus exclude two potential mechanisms that could link α-cell secretion activity to increased β-cell secretion.

We have thus added the following text at the end of page 9 – top of page 10: "To determine whether this could be due to increased glucagon secretion from alpha cells or to increased processing of preproglucagon into, and secretion of GLP-1 from alpha cells, we performed insulin secretion experiments in the presence of the glucagon receptor antagonist L-168,049 and of the GLP-1R antagonist exendin(9-39). No inhibitory effects on glucose stimulated insulin secretion by islets from *αGckKO* mice could be observed in these conditions (Supplementary Fig. 7a,b) and qRT-PCR analysis showed that the level of *Pcsk1/3* was not increased in *αGckKO* α-cells (Supplementary Fig. 7c).

We also modified the last sentence of the first paragraph of page 12 as follows (red):" This β-cell adaptation may result from direct α-cell to β-cell communication, other than through glucagon or GLP-1 signaling, or may be indirect,…"

Due to the quality of the manuscript, I have only two specific minor comments:

1. Abstract: „…over time, induces a pre-diabetic phenotype". The wording "pre-diabetic" is not completely accurate: because the precise role alpha cell glucokinase in diabetes is not elucidated, it remains to be shown whether observed changes would really contribute to a diabetic phenotype.

We thank the reviewer for the suggestion. However, we used the term "pre-diabetic" on the basis of the metabolic phenotype developed by adult *αGckKO* mice – i.e. glucose intolerance and altered insulin secretion – and not arguing that alpha-cell Gck is involved in diabetes onset.

2. Typo: Discussion Line 186: glucose intolerance (not tolerance).

This has been corrected.

Reviewer #1 (Remarks to the Author):

All issues were addressed. Very nice paper.

Reviewer #2 (Remarks to the Author):

No comments

Reviewer #3 (Remarks to the Author):

No comment

Reviewer #4 (Remarks to the Author):

The responses are convincing
